# Virgin Olive Oil Ranks First in a New Nutritional Quality Score Due to Its Compositional Profile

**DOI:** 10.3390/nu15092127

**Published:** 2023-04-28

**Authors:** Aída García-González, Angelica Quintero-Flórez, María-Victoria Ruiz-Méndez, Javier S. Perona

**Affiliations:** 1 Department of Molecular Biology and Biochemical Engineering, University of Pablo de Olavide, 41013 Seville, Spain; 2 Department of Food and Health, Instituto de la Grasa-CSIC, 41013 Seville, Spain; perona@ig.csic.es; 3 Department of Preventive Medicine and Public Health, University of Seville, 41009 Seville, Spain; 4 Department of Characterization and Quality of Lipids, Instituto de la Grasa-CSIC, 41013 Seville, Spain

**Keywords:** bioactive compounds, fat, fatty acids, nutrients, nutritional quality, oil, score

## Abstract

Dietary oils play a crucial role in maintaining a healthy diet. However, with the increasing number of oils available, it became a challenging task for food producers and consumers to select the best oil for their needs. In this work, an easy-to-understand nutrition quality score was created, using a model that included beneficial lipid compounds criteria according to the dietary recommendations published by international food and health organizations. The algorithm assigned points for each component of the model considering their content in each particular oil. The points were added up and the fats and oils were classified by the corresponding percentile. As a result, among the 32 edible oils that were evaluated, virgin olive oil ranked first with a score of 100. All plant oils, except for margarine and coconut oil, ranked above the 50th percentile. Receiver–operator curves and regression models showed that saturated fatty acids may be able to predict the score, and thus, the nutritional quality of the oils. In conclusion, the proposed nutritional quality score would promote healthy and nutritious food options for consumers and would provide food producers with a valuable tool to select high-quality oils for their products, ensuring that they meet the nutritional requirements.

## 1. Introduction

Dietary oils are essential ingredients in many food products and are widely used for cooking and food processing. In recent years, there was an increase in the demand for edible oils due to the growth of the food industry and the changing dietary habits of consumers [1]. A variety of edible oils are produced from various sources such as nuts, seeds, and fruits, and their nutritional quality can vary greatly [2].

Currently, there is a lack of a standardized ranking system for edible oils based on their nutritional quality. Producers and consumers are often unaware of the nutritional differences among oils, making it difficult for them to make informed decisions [3]. This results in the production and consumption of oils with lower nutritional quality, which can have negative impacts on public health. Therefore, developing a score for edible oils based on their nutritional quality is of utmost importance, as it would allow consumers to make informed decisions about the oils they use and would encourage producers to improve the nutritional quality of their oils.

For the moment, there is no universally accepted score to rank edible oils according to their nutritional value. The smoke point score, which measures the smoke point of an oil and classifies edible oils accordingly, is calculated according to the official American Oil Chemists’ Society Cc 9a-48 method [4]. However, this score does not consider the composition of the oils and the recommendations of international organizations responsible for dietary recommendations, such as the Food and Agriculture Organization (FAO)/World Health Organization (WHO) [5,6], European Food Safety Authority (EFSA) [7,8], or the United States Department of Agriculture (USDA)/Food and Drug Administration (FDA) [9]. On the other hand, the American Heart Association publishes recommendations for oils based on their fatty acid composition, which can also be used as a guide for ranking edible oils based on their nutritional quality, but never created a score [10]. 

Recently, Zhao et al. [11] published a model for the evaluation of the nutritional quality of 13 edible vegetable oils commonly used in China. Employing seven evaluation criteria, including saturated fatty acids (SFA), unsaturated fatty acids (UFA), and vitamin E, they found that the highest score was obtained for peony seed oil, while the lowest score belonged to palm oil. However, some components of the oils were out of the analysis, such as polyphenols. In addition, the model relied on the French and Chinese Dietary Reference Intake recommendations only.

In the present study, we propose a new score based on the Dietary Reference Intake provided by the FAO/WHO [5,6], EFSA [7,8], and USDA/FDA [9], as well as health claims approved by the EFSA [12,13] and USDA/FDA [14,15] in 32 dietary plant oils and animal fats. A ranking system based on nutritional quality would provide a basis for evaluating the health benefits of the oils. This would be beneficial for public health as it would promote the use of oils with higher nutritional quality, reducing the risk of chronic diseases. Furthermore, a ranking system would promote the development of new and healthier oils, contributing to the growth of the food industry.

## 2. Materials and Methods

### 2.1. Recommended Dietary Intakes and Health Claims

To design a new nutritional quality score, the dietary reference values (RDVs) for the intake of nutrients contained in edible oils (total fat, fatty acids, tocopherols, and phytoestrogens) published by the FAO/WHO [5,6], EFSA [7,8], and USDA/FDA [9], as well as health claims approved by the EFSA [12,13] and USDA/FDA [14,15], were compiled for the population over 18 years of age (Table 1). We found RDV for SFA, linoleic (18:2 *n*-6) and α-linolenic (18:3 *n*-3) acids, eicosapentaenoic (EPA) plus docosahexaenoic (DHA) acids, trans fatty acids (TFA), and tocopherols. Among these international organizations, only the FAO/WHO provides RDV for SFA as well as TFA, whereas the EFSA and FDA recommend that the consumption of these fatty acids be as low as possible. Regarding linoleic and α-linolenic acids, all three organizations give RDV in the form of percentages of daily energy intake. For the long-chain fatty acids EPA and DHA acids, and also for tocopherols, values are given as daily amounts in mg by the FAO and EFSA, but not USDA. 

In addition, we found approved health claims for hydroxytyrosol and plant sterols by the EFSA, as well as oleic acid (18:1 *n*-9) by the FDA. The health claims published for hydroxytyrosol only affect olive oils, as this phenolic is found in olive fruits and leaves and is given as mg in a specific amount of olive oil. In contrast, plan sterols are found ubiquitously in the plant kingdom, hence the EFSA recommends a specific amount in grams from any plant-derived food. Oleic acid is present in plants and animals, but the FDA recognizes its health benefits only for those fats and oils with a concentration above 70%.

### 2.2. Nutritional Composition of Edible Oils and Fats

The composition in SFA, oleic acid, linoleic acid, α-linolenic acid, EPA plus DHA, TFA, tocopherols, hydroxytyrosol, and phytosterols were gathered for 32 edible oils and fats (for references see Supplementary Tables S1 and S2). The lowest and highest concentrations of each nutrient and bioactive component were considered, and the mean value of these concentrations was used for calculations (for more information see Supplementary Tables S1 and S2). 

### 2.3. Algorithm and Calculation of the Nutritional Quality Score

Since the RDVs were given in different manners by the FAO, EFSA, USDA, and FDA, values were normalized for a daily energy intake of 2000 kcal. Then, the contribution of each component was calculated for a fat intake of 35% (700 kcal), which is the highest fat intake recommended by the FAO. Subsequently, the concentration of each component per 100 g was calculated, so fats and oils could be compared (Table 1).

According to the RDV, points between −5 and +3 where given for each component. Negative points were assigned to those components with maximum recommended values, and positive points were assigned to those with minimum recommended values and health claims. Thus, for SFA contents above 10 g/100 g −3 points were assigned, as well as 0 points below that value. Since the current evidence shows that TFA intake is more hazardous for health than SFA, contents above 1 g/100 g were penalized with −5 points and 0 points below that value. For linoleic acid, contents above 6 g/100 g received +3 points, while those below that value received −3 points. Similarly, contents of α-linolenic acid received +2 or −2 points when they were above or below 0.5 g/100 g, and the contents of EPA + DHA received +3 or −3 points when they were above or below 0.11 g/100 g. Points were summed up for all oil and fat, and the resulting values were normalized by subtracting the lowest value (−14). The nutritional quality resulting scores were established by calculating the corresponding percentiles, so the highest score was 100 and the lowest score was 0. 

### 2.4. Statistical Analysis

The ROC curves were calculated to evaluate the abilities of the components of oils and fats to predict the nutritional quality score. Cut-off points were proposed after calculation of the Youden’s Index (sensitivity+ specificity − 1). The normality of the distribution for the different variables was assessed using the Kolmogorov–Smirnov test. Correlations were analyzed using the Spearman test. EPA and DHA, as well as hydroxytyrosol and TFA, were not included in the ROC calculations and correlation assessments, as they were not present in most oils and fats. GraphPad Prism 6.0 was used to perform statistical analyses. Statistical significance was defined as *p* < 0.05.

## 3. Results

### 3.1. Nutritional Quality Score of Edible Oils and Fats

VOO showed the highest value for the nutritional quality score, with 8 points, which corresponded to a score of 100 (Table 2). All plant oils, except for margarine and coconut oil, scored above the 50th percentile. In fact, these two fats showed the lowest score, with 14 and 0 score points, respectively (−11 and −14 points). Fats derived from fish, i.e., salmon, sardine, and herring, received points above the 68th percentile. In contrast, the rest of fats derived from animals, i.e., beef tallow, lard, and butter, scored below the 50th percentile.

**Figure 1:**
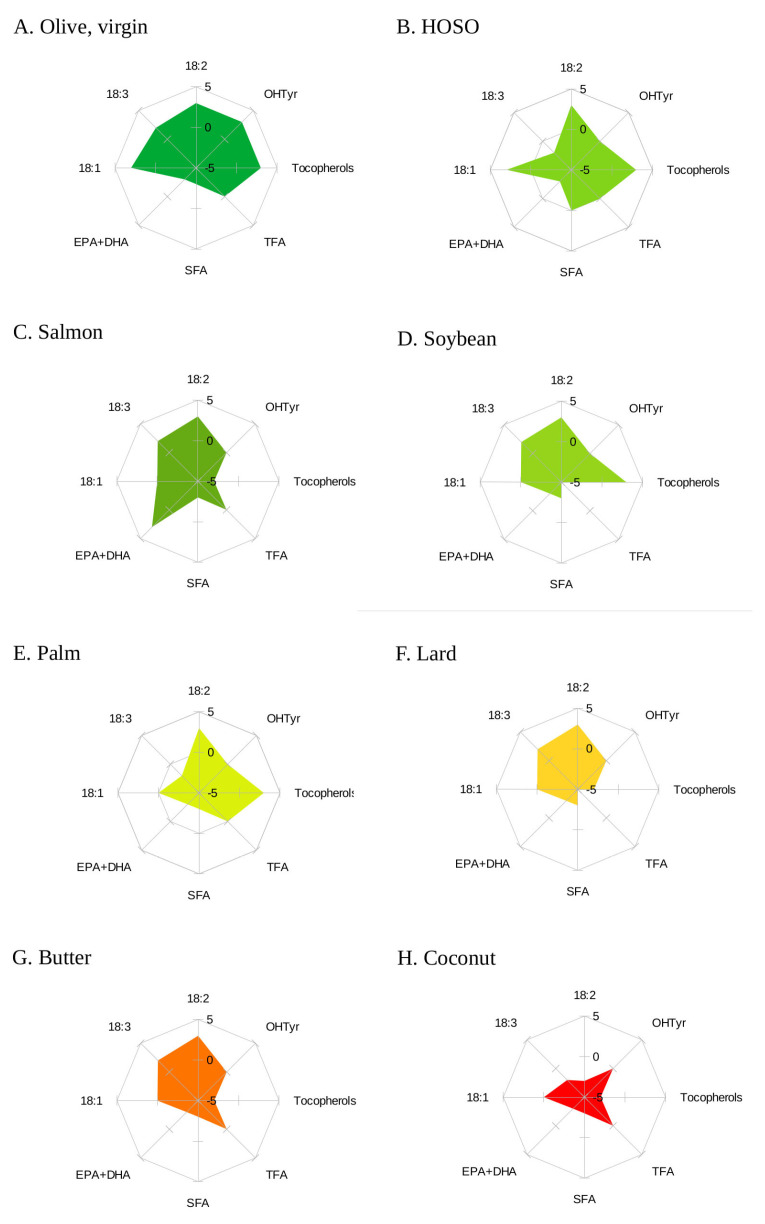
Radar chart for the score points assigned to SFA, oleic acid (18:1), linoleic acid (18:2), α-linolenic acid (18:3), eicosapentaenoic and docosahexaenoic acids (EPA + DHA), trans fatty acids (TFA), hydroxytyrosol (OHTyr), tocopherols and phytosterols in VOO.

The contribution of each component of the oils and fats used in the model to the overall score is shown graphically in Figure 1.

### 3.2. Correlation between Components of Oils and Fats and the Nutritional Quality Score

Only SFA and phytosterols correlated significantly with the nutritional quality score (Table 3). While the SFA correlation coefficient was negative (−0.503), that of phytosterols was positive (0.357), which implies that higher SFA contents and lower phytosterol contents are related to higher scores. Oleic, linoleic, and α-linolenic acids, together with tocopherols, did not correlate with the nutritional quality score. 

**Figure 2:**
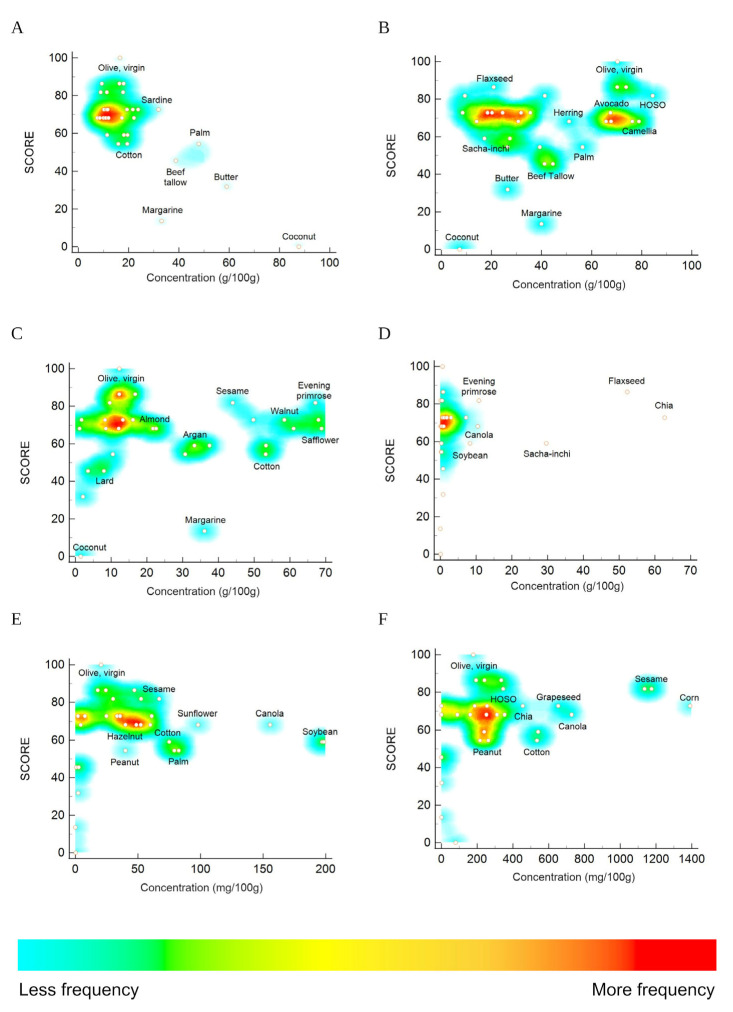
Heat map charts showing the content in SFA (**A**), oleic acid (**B**), linoleic acid (**C**), α-linolenic acid (**D**), tocopherols (**E**), and phytosterols (**F**) of the edible oils evaluated. Colors show the areas with more or less (light blue) oils and fats with similar scores and concentrations of a particular component expressed as frequency.

Figure 2 shows heat map charts showing the content in SFA, oleic acid, linoleic acid, α-linolenic acid, tocopherols, and phytosterols of the edible oils evaluated. The correlation of the SFA content and the score, considering the frequency of each oil, is clearly observed. Most oils presented SFA contents below 20 g/100 g, which conduced to scores above 50. In other words, lower scores were associated with higher SFA contents, which corresponded to beef tallow, lard, butter, and coconut oil. The other components of the fats and oils used in the development of the score were unevenly distributed in the charts, not showing a clear pattern.

### 3.3. Prediction of the Nutritional Quality Score

ROC curves were calculated for SFA, oleic acid, linoleic acid, α-linolenic acid, tocopherols, and phytosterols in order to assess the possibility that these components might predict the nutritional quality score of the fats and oils (Table 4). SFA and α-linolenic acid showed an area under the curve (AUC) values above 0.9, which suggests that these fatty acids have a high predictive value for the score. In contrast, oleic acid, tocopherols, and phytosterols showed the lowest AUC values (below 0.8), despite the latter correlated with the score (Table 3). The calculated cut-off point for SFA was 42.2, which implies that oils and fats with SFA contents above that value have a poor nutritional quality score. Among the oils and fats employed in this study, beef tallow, butter, palm oil, and coconut oil showed SFA contents above 42.2 g/100 g, while lard was slightly below that figure. The cut-off point calculated for α-linolenic acid was 12.2, which suggests that above that concentration, the score is favorable. Chia, flaxseed, sacha-inchi, walnut, and canola oils presented average concentrations above 12.2 g/100 g. The mean value calculated for soybean oil was 12.1 g/100 g.

## 4. Discussion

The use of a score-based system to rank dietary oils based on their nutritional value is important, as it enables food producers and consumers to make informed decisions when selecting and purchasing oils for their products and meals. This would ensure that high-quality and nutritious oils are used in food production and consumed by individuals. The score developed here was based on the RDV of international institutions, such as the FAO/WHO [5,6], EFSA [7,8] and USDA/FDA [9], as well as health claims approved by the EFSA [12,13] and USDA/FDA [14,15].

The top-ranking oil in the proposed score was VOO, with 100 points due to its unique compositional profile. One of the factors contributing to the positive score for VOO was its high oleic acid content (55–83 g/100 g, according to the IOC commercial standard ref), which is associated with a reduced risk of coronary heart disease. The FDA approved health claims that suggest the consumption of oils high in oleic acid may help reduce the risk of heart disease when used in place of oils high in saturated fat [16]. The claims also emphasize that oleic acid-containing oils should not increase daily caloric intake and are intended for oils with oleic acid contents above 70% (70 g/100 g). The EU also established a list of permitted health claims for oleic acid-rich oils when replacing saturated fats in the maintenance of normal blood cholesterol levels. However, it does not state the threshold at which to consider a fat as “high-oleic”. VOO also contains other health-promoting compounds, such as linoleic acid, α-linolenic acid, tocopherols, and hydroxytyrosol, which contribute positively to the score. According to the OIC standard, the linoleic acid content should range between 2.5 and 21.0 g/100 g. The mean value used in the present study for the content of linoleic acid in VOO was 12.3, which guaranteed 3 points in the score. Similarly, the α-linolenic content in this oil was above the threshold, adding 2 points more to the score, and the tocopherol content contributed 3 more points. In contrast, the phytosterol content was not high enough to add up points, and VOO received negative points due to its lack of long-chain polyunsaturated fatty acids (PUFA) of the n-3 series and the presence of SFA. According to the IOC commercial standard, the SFA content of VOO ranges from 9 to 25 g/100 g. The mean content used to evaluate VOO in the present study was 16.6, which is higher than the concentration used as criterion (9 g/100 g). 

Finally, it is noteworthy that VOO is the only fat that contains hydroxytyrosol in enough of a concentration to add points to the score. Hydroxytyrosol and its derivatives also have a health claim approved by the EFSA for olive oil, as a cause and effect relationship was established between the consumption of olive oil and the protection of LDL particles from oxidative damage [17]. However, only VOO, and not common and pomace olive oils, presented concentrations of polyphenols high enough to reach the minimum concentration stated in the claim (5 mg per 20 mL of oil). Common and pomace olive oils, which share the same fatty acid composition with VOO, did not present concentrations of polyphenols high enough to add points in the score. It is important to note that these olive oils are submitted to a refining process that removes some of the beneficial minor components present in VOO.

Olive oil, and particularly VOO, is widely studied for its potential health benefits, particularly in the prevention of cardiovascular disease. A meta-analysis conducted in 2019 showed that high-phenolic extra virgin olive oil (VOO) was effective in reducing LDL-cholesterol, systolic blood pressure, and oxidized LDL-cholesterol levels [18]. In addition, a systematic review by the USDA reported that a Mediterranean-style diet, including olive oil, was associated with a reduced risk of all-cause mortality in nearly all studies examined [19]. A part of the health benefits of VOO is attributed to its high content of polyphenols, which were shown to improve plasma concentrations of malondialdehyde, oxidized LDL, total cholesterol, and HDL cholesterol in clinical trials [20]. These effects appear to be maintained even when the oil is used for cooking [19]. However, some researchers noted that the evidence is still limited and that more large clinical trials are needed to confirm these benefits [21]. While the evidence is still emerging, the consumption of VOO as part of a healthy diet showed promise in promoting cardiovascular health and reducing the risk of all-cause mortality.

Flaxseed oil, which ranked second after VOO and ahead of the other olive oils (common and pomace), is known for its high content of α-linolenic acid, similarly to chia and sacha-inchi oils. Flaxseed oil is also richer in tocopherols and has a lower level of SFA compared to other oils. Numerous systematic reviews and meta-analyses investigated the health benefits of consuming flaxseed oil. A recent systematic review of randomized clinical trials in patients with metabolic syndrome and related disorders reported that flaxseed oil consumption significantly reduced systolic blood pressure, but not diastolic blood pressure [22]. Additionally, Musazadeh et al. [23] conducted a meta-analysis that aimed to determine the impact of flaxseed oil on biomarkers of oxidative stress, and found a significant decrease in malondialdehyde levels and an increase in total antioxidant capacity levels, indicating that it may be useful in reducing oxidative stress and strengthening the antioxidant defense system in humans.

The ranking of vegetable oils based on their content in the components studied revealed that all oils, except for margarine and coconut oil, scored above 50. One notable case was palm oil, which was previously associated with adverse cardiovascular health outcomes, as it was reported that it increased LDL-cholesterol levels compared to vegetable oils low in saturated fat [24]. However, more recent systematic reviews and meta-analyses showed only modest increases in serum lipids [25] or no increase at all when compared to oils rich in UFA [26].

After calculating percentiles, coconut oil received the lowest score of 0 points due to its high SFA content, resulting in low concentrations of UFA. In addition, the presence of phytosterols and tocopherols was very low, which contributed greatly to the low punctuation. Despite claims made by consumers on social media regarding the health benefits of coconut oil [27], recent meta-analyses showed that consumption of this oil increases serum lipid concentrations more than oils rich in monounsaturated fatty acids (MUFA) and PUFA [28,29,30], which is comparable to that of animal fats rich in SFA [31].

Margarine was strongly linked to increased risk of cardiovascular disease due to its content in TFA. As a result, the WHO recommended eliminating industrially produced TFA, and several countries implemented regulations to reduce or ban the use of partially hydrogenated fat [32]. As a consequence, the TFA concentration in margarines varies greatly depending on their origin of production. The nutritional quality score of margarine included products with TFA content ranging from 0.1 to 21.7 g/100 g, with a mean value of 10.9. Therefore, this high TFA content contributed to a low score value for margarine and a low position in the ranking. However, margarines are frequently enriched with vitamin E and/or phytosterols, which could contribute to a higher score for this food.

In terms of nutritional quality, the score of animal fats was highly variable. Fish fats, due to their high content in n-3 PUFA, ranked best, occupying positions 12 (salmon) to 19 (herring) in the ranking. In contrast, lard, beef tallow, and butter ranked at the bottom of the classification, just above coconut oil. The low score obtained by animal fats other than in fish was mainly due to their high content in SFA, which is associated with elevated levels of LDL-cholesterol [33,34,35]. Despite some controversy about the role of SFA in cardiovascular health [36], limiting the intake of SFA to less than 6% and 10% of daily energy is recommended by the American Heart Association [37] and the FAO/WHO [5,6], respectively (Table 2). We found that SFA strongly correlated with the nutritional quality score and provided a high AUC value in ROC curves, indicating that the SFA content of a fat or oil can be used to predict the score. 

The current study has several strengths and limitations. An important strength is that the score was developed by considering the RDV and health claims of four important international food and health organizations (FAO/WHO, EFSA, USDA, and FDA). Secondly, the model included nine criteria, including fatty acids, micronutrients, and bioactive compounds, for which nutritional dietary recommendations or health claims were available. Moreover, the study included 32 fats and oils, both of plant and animal origin, which is a larger sample size than the previous study by Zhao et al. [11], the only authors that previously attempted to elaborate a score to rank vegetable oils according to their nutritional quality, to the best of our knowledge. These authors, included only 13 vegetable oils and did not differentiate between VOO and common and pomace olive oils, just as other popular nutritional ratings, such as Nutri-Score [38]. However, the study also has limitations. For instance, the score did not consider non-compositional parameters of oils, such as their smoke point and resistance to oxidation, which could influence their nutritional quality over time. Additionally, despite the large sample size of edible oils and fats used, the score could be enhanced by increasing the number of oils evaluated. Finally, the composition of some oils is highly variable, which could lead to changes in the score.

## 5. Conclusions

In conclusion, we propose a score-based system to rank dietary oils based on their nutritional value, according to the RDV and health claims by the FAO/WHO, EFSA, USDA, and FDA, which can be useful for both food producers and consumers to make informed decisions. Using this score, VOO ranked first with 100 points, thanks to its compositional profile, which includes high levels of oleic acid and the presence of linoleic acid, α-linolenic acid, and tocopherols. Importantly, VOO contains a number of other compounds of interest in health, among which, hydroxytyrosol has an approved health claim by the EFSA. Future research is still needed to confirm the benefits of VOO, flaxseed, and other dietary fats and oils on health, but the score-based system proposed in this study can be useful in guiding food producers and consumers towards more nutritious choices.

## Figures and Tables

**Table 1:** Dietary reference values according to the FAO, USDA, and EFSA, including approved claims for hydroxytyrosol and plant sterols.

Nutrient ^1^	FAO/WHO [5,6]	USDA/FDA [9]	EFSA [7,8]	2000 Kcal	750 Kcal	100 g Fat/Oil
SFA (g/day)	<10% E	ALAP	ALAP	<22.2	<7.0	<9.0
18:1		>70%				>70
18:2 (g/day)	2–3% E	5–10% E	4% E	3.6–13.5	1.3–4.7	1.6–6.1
18:3 (g/day)	0.5–2% E	0.6–1.2% E	0.5% E	1.1–4.4	0.4–1.7	0.5–2.2
EPA + DHA (g/day)	0.25–2.00		0.25	0.25–2.00	0.09–0.70	0.11–0.90
TFA (mg/day)	<1% E	ALAP	ALAP	<2.2	<0.7	<0.9
Tocopherols (Vit. E) (mg/day)	7.5–10	12–15	11–13	7.5–15.0	2.6–5.3	3.4–6.7
Hydroxytyrosol			5 mg/20 mL [13]			25 mg/100 mL
Phytosterols (g/day)			1.5–3.0 [12]	1.5–3.0	0.5–1.1	0.7–1.4

^1^ For the calculation, a daily energy intake of 2000 kcal was taken into account and the contribution of each nutrient was calculated for a fat intake of 35% (700 kcal), as recommended by the FAO. Then, the concentration of each nutrient per 100 g of fat was calculated. For oleic acid, a claim approved by the FDA was considered. For hydroxytyrosol and sterols, claims approved by the EFSA were considered. ALAP, as low as possible; E, energy; and Vit. E, vitamin E.

**Table 2:** Sum of points, normalized sum, and nutritional quality score of the oils.

Rank	Oil	Sum	Normalized Sum ^1^	Score
1	Olive, virgin	8	22	100
2	Flaxseed	5	19	86
3	Olive, common	5	19	86
4	Olive, pomace	5	19	86
5	Evening primrose	4	18	82
6	Sunflower, high-oleic	4	18	82
7	Sesame	4	18	82
8	Avocado	2	16	73
9	Chia	2	16	73
9	Corn	2	16	73
11	Grapeseed	2	16	73
12	Salmon	2	16	73
13	Sardine	2	16	73
14	Walnut	2	16	73
15	Almond	1	15	68
16	Camelia	1	15	68
17	Canola	1	15	68
18	Hazelnut	1	15	68
19	Herring	1	15	68
20	Safflower	1	15	68
21	Sunflower	1	15	68
22	Argan	−1	13	59
23	Sacha-inchi	−1	13	59
24	Soybean	−1	13	59
25	Cotton	−2	12	55
26	Palm	−2	12	55
27	Peanut	−2	12	55
28	Beef tallow	−4	10	45
29	Lard	−4	10	45
30	Butter	−7	7	32
31	Margarine	−11	3	14
32	Coconut	−14	0	0

^1^ Sums were normalized by subtracting the lowest area (coconut oil) to the sum of each oil. The score was calculated as the percentile of the normalized sums.

**Table 3:** Regression coefficients for the association of nutrient content in the oils and the score.

Nutrient	Spearman’s Rho Coefficient	*p*	CI (95%)
SFA	−0.503	0.0034	−0.724 to −0.187
18:1	0.190	0.3067	−0.176 to 0.510
18:2	0.158	0.3892	−0.202 to 0.480
18:3	0.326	0.0684	−0.025 to 0.606
Tocopherols	0.039	0.8329	−0.314 to 0.382
Phytosterols	0.357	0.0449	0.009 to 0.628

**Table 4:** AUC, optimal cut-off point, sensitivity, specificity, and Youden Index in the ROC curve analysis of six criteria used in the score.

Nutrient	AUC	SD ^1^	95% CI ^2^	*p* Value	Cut-off	Sensitivity	Specificity	Youden Index
SFA	0.906	0.045	0.818–0.995	<0.0001	42.2	0.906	0.875	0.781
18:1	0.770	0.062	0.648–0.892	0.0002	52.8	0.844	0.677	0.521
18:2	0.892	0.042	0.809–0.975	<0.0001	53.9	0.844	0.844	0.688
18:3	0.954	0.032	0.891–1.016	<0.0001	12.2	0.969	0.875	0.844
Tocopherols	0.718	0.070	0.582–0.855	0.0027	67.6	0.656	0.681	0.338
Phytosterols	0.777	0.072	0.637–0.918	0.0001	76.4	0.781	0.781	0.563

^1^ SD, standard deviations; ^2^ CI, coefficient interval.

## Data Availability

Data are available upon request.

## Supplementary Materials

The following supporting information can be downloaded at: https://www.mdpi.com/article/10.3390/nu15092127/s1, Table S1: Lowest, highest and mean content of the fatty acids included as criteria for the developing of the nutritional quality score of dietary oils (g/100 g); Table S2: Lowest, highest and mean content of trans fatty acids, tocopherols and sterols as criteria for the developing of the nutritional quality score of dietary oils.
