# Supplementary material for: Virgin Olive Oil Ranks First in a New Nutritional Quality Score Due to Its Compositional Profile
Source: Nutrients. 2023 Apr 28;15(9):2127. doi: 10.3390/nu15092127 (PMC10180740; doi:10.3390/nu15092127)
Supplement: Supplementary file 1 [file nutrients-15-02127-s001.zip › References for Supplementary Tables S1 and S2.pdf]

References for Supplementary Tables S1 and S2

1. Orsavova, J.; Misurcova, L.; Vavra Ambrozova, J.; Vicha, R.; Mlcek, J. Fatty acids composition of vegetable oils and its contribution to dietary energy intake and dependence of cardiovascular mortality on dietary intake of fatty acids. *Int. J. Mol. Sci.* **2015**, 16(6), 12871-12890. DOI:10.3390/ijms160612871

2. Yang, R.; Zhang, L.; Li, P.; Yu, L.; Mao, J.; Wang, X.; Zhang, Q. A review of chemical composition and nutritional properties of minor vegetable oils in China. *Trends Food Sci. Technol.* 2018, 74, 26-32. DOI:10.1016/j.tifs.2018.01.013

4. Ouzir, M.; Bernoussi, S.E.; Tabyaoui, M.; Taghzouti, K. Almond oil: A comprehensive review of chemical composition, extraction methods, preservation conditions, potential health benefits, and safety. Compr. *Rev. Food Sci. Food Saf.* **2021**, 20(4), 3344-3387. DOI: 10.1111/1541-4337.12752

3. Ramadan, M.F. *Fruit oils: chemistry and functionality*, 1st ed.; Springer: Switzerland, 2019. DOI:10.1007/978-3-030-12473-1

5. Bennani, H.; Drissi, A.; Giton, F.; Kheuang, L.; Fiet, J.; Adlouni, A. Antiproliferative effect of polyphenols and sterols of virgin argan oil on human prostate cancer cell lines. *Cancer Detect. Prev.* **2007**, 31 (1), 64-69. DOI: 10.1016/j.cdp.2006.09.006

6. Gharby, S.; Charrouf, Z. Argan oil: chemical composition, extraction process, and quality control. *Frontiers in Nutr.* **2020**, 8, 1251. DOI: 10.3389/fnut.2021.804587

7. Menni, H.B.; Belarbi, M.; Menni, D.B.; Bendiab, H.; Kherraf, Y.; Ksouri, R.; Visioli, F. et al. Anti-inflammatory activity of argan oil and its minor components. *Int. J. Food Sci. Nutr.* **2020**, 71(3), 307-314. DOI: 10.1080/09637486.2019.1650005

8. Miklavčič, M.B.; Taous, F.; Valenčič, V.; Elghali, T.; Podgornik, M.; Strojnik, L.; Ogrinc, N. Fatty acid composition of cosmetic argan oil: provenience and authenticity criteria. *Molecules* **2020**, 25(18), 4080. DOI: 10.3390/molecules25184080

9. Simões, T.; Ferreira, J.; Lemos, M.F.; Augusto, A.; Félix, R.; Silva, S.F.; Tecelão, C. Argan oil as a rich source of linoleic fatty acid for dietetic structured lipids production. *Life* **2021**, 11(11), 1114. DOI: 10.3390/life11111114

10. USDA. Food Data Central. Available online: https://fdc.nal.usda.gov/fdc-app.html#/

11. Woolf, A.; Wong, M.; Eyres, L.; McGhie, T.; Lund, C.; Olsson, S.; Requejo-Jackman, C.; et al. Avocado oil. In *Gourmet and health-promoting specialty oils;* Robert A. Moreau, R.A., Kamal-Eldin, A.; AOCS Press, 2009; pp. 73-125.

12. Stolp, L.J.; Kodali, D.R; Naturally occurring high-oleic oils: Avocado, macadamia, and olive oils. In *High Oleic Oils*; AOCS Press; 2022; pp. 7–52. DOI: 10.1016/B978-0-12-822912-5.00003-4

13. CODEX, S. 211. Standard for named Animal Fats. 1999.

14. Turchini, G.M.; Torstensen, B.E.; Ng, W.K. Fish oil replacement in finfish nutrition. *Rev. Aquac.* **2009**, 1(1), 10-57. DOI: 10.1111/j.1753-5131.2008.01001.x

15. SOJÁK, I.F.W.L.L. Fatty acid composition of summer and winter cows' milk and butter. *J. Food Nutr. Res.* **2010**, 49(4), 169-177.

16. Cheng, X.; Yang, T.; Wang, Y.; Zhou, B.; Yan, L.; Teng, L.; Zhang, D.; et al. New method for effective identification of adulterated Camellia oil basing on Camellia oleifera-specific DNA. *Arabian J. Chem.* **2018**, 11(6), 815-826. DOI: 10.1016/j.arabjc.2017.12.025

17. Shi, T.; Wu, G.; Jin, Q.; Wang, X. Camellia oil authentication: A comparative analysis and recent analytical techniques developed for its assessment. A review. *Trends Food Sci. Technol.* **2020**, 97, 88-99. DOI: 10.1016/j.tifs.2020.01.005

18. CODEX Alimentarius. Codex standard for named vegetable oils. Codex stan, 210, 1999, 1-13.
19. Zambiazi, R.C.; Przybylski, R.; Zambiazi, M.W.; Mendonça, C.B. Fatty acid composition of vegetable oils and fats. *Bol. Cent. de Pesqui. de Process. de Aliment.* **2007**, 25(1). DOI:10.5380/cep.v25i1.8399

20. Kostik, V.; Memeti, S.; Bauer, B. Fatty acid composition of edible oils and fats. *J. Hyg. Eng. Des.* **2013**, 4, 112-116.

21. Ghazani, S. M.; Marangoni, A.G. Minor components in canola oil and effects of refining on these constituents: A review. *J. Am. Oil Chem. Soc.* **2013**, 90(7), 923-932. DOI:10.1007/s11746-013-2254-8

22. Ghafoor, K.; Aljuhaimi, F.; Özcan, M.M., Uslu, N.; Hussain, S.; Babiker, E.E.; Fadimu, G. Effects of roasting on bioactive compounds, fatty acid, and mineral composition of chia seed and oil. *J. Food Process. Preserv.* **2018**, 42(10). DOI: 10.1111/jfpp.13710

23. Abad, A.; Shahidi, F. Compositional characteristics and oxidative stability of chia seed oil (Salvia hispanica L). *Food Prod. Process. Nutr.* **2020**, 2(1), 1-8. DOI:10.1186/s43014-020-00024-y

24. Rokosik, E.; Dwiecki, K.; Siger, A. Nutritional quality and phytochemical contents of cold pressed oil obtained from chia, milk thistle, nigella, and white and black poppy seeds. *Grasas y Aceites* **2020**, 71(3), e368-e368. DOI: 10.3989/gya

25. Chowdhury, K.; Banu, L.A.; Khan, S.; Latif, A. Studies on the fatty acid composition of edible oil. *Bangladesh J.Ssci.Ind.Rres.* **2007**, 42(3), 311-316. DOI:10.3329/bjsir.v42i3.669

26. Dorni, C.; Sharma, P.; Saikia, G.; Longvah, T. Fatty acid profile of edible oils and fats consumed in India. *Food Chem.* **2018**, 238, 9-15. DOI: 10.1016/j.foodchem.2017.05.072

27. Abu, E.H.; Mahmoud, A.A.T. Effect of refining process on the quality characteristics of soybean and cotton seed oils. *Int. J. Curr. Microbiol. App. Sci.* **2017**, 6(1), 207-222. DOI:10.20546/ijcmas.2017.601.026

28. Christie, W.W. The analysis of evening primrose oil. *Ind. Crops Prod.* **1999**, 10(2), 73-83. DOI: 10.1016/S0926-6690(99)00013-8

29. Eskin, N.M. Borage and evening primrose oil. *Eur. J. Lipid Sci. Technol.* **2008**, 110(7), 651-654. DOI: 10.1002/ejlt.200700259

30. Zhao, B.; Gong, H.; Li, H.; Zhang, Y.; Deng, J.; Chen, Z. Fatty acid, triacylglycerol and unsaponifiable matters profiles and physicochemical properties of Chinese evening primrose oil. *J. Oleo Sci.* **2019**, 68(8), 719-728. DOI: 10.5650/jos.ess19091

31. Pan, F.; Li, Y.; Luo, X.; Wang, X.; Wang, C.; Wen, B.; Liu, B.; et al. Effect of the chemical refining process on composition and oxidative stability of evening primrose oil. *J. Food Process. Preserv.* **2020**, 44(10), e14800. DOI: 10.1111/jfpp.14800

32. Khattab, R.Y.; Zeitoun, M.A. Quality evaluation of flaxseed oil obtained by different extraction techniques. *Food Sci. Technol.* **2013**, 53(1), 338-345. DOI: 10.1016/j.lwt.2013.01.004

33. Hashempour-Baltork, F.; Torbati, M.; Azadmard-Damirchi, S.; Savage, G.P. Quality properties of sesame and olive oils incorporated with flaxseed oil. *Adv. Pharm. Bull.* **2017**, 7(1), 97. DOI: 10.15171/apb.2017.012

34. Lutterodt, H.; Slavin, M.; Whent, M.; Turner, E.; Yu, L.L. Fatty acid composition, oxidative stability, antioxidant and antiproliferative properties of selected cold-pressed grape seed oils and flours. *Food Chem.* **2011**, 128(2), 391-399. DOI: 10.1016/j.foodchem.2011.03.040

35. Garavaglia, J.; Markoski, M.M.; Oliveira, A.; Marcadenti, A. Grape seed oil compounds: Biological and chemical actions for health. *Nutr. Metab. Insights* **2016**, 9, NMI-S32910. DOI: 10.4137/NMI.S32910

36. Martin, M.E.; Grao-Cruces, E.; Millan-Linares, M.C.; Montserrat-De la Paz. Grape (Vitis vinifera L.) seed oil: A functional food from the winemaking industry. *Foods* **2020**, 9(10), 1360. DOI: 10.3390/foods9101360

37. Benitez-Sánchez, P.L.; León-Camacho, M.; Aparicio, R. A comprehensive study of hazelnut oil composition with comparisons to other vegetable oils, particularly olive oil. *Eur. Food Res. Technol.* **2003**, 218(1), 13-19. DOI:10.1007/s00217-003-0766-4

38. Karabulut, I.; Topcu, A.; Yorulmaz, A.; Tekin, A.; Ozay, D.S. Effects of the industrial refining process on some properties of hazelnut oil. *Eur. J. Lipid Sci. Technol.* **2005**, 107(7-8), 476-480. DOI: 10.1002/ejlt.200501147

39. Aidos, I.; Lourenclo, S.; Van der Padt, A.; Luten, J.B.; Boom, R.M. Stability of crude herring oil produced from fresh byproducts: influence of temperature during storage. *J. Food Sci.* **2002**, 67(9), 3314-3320. DOI: 10.1111/j.1365-2621.2002.tb09585.x

40. Kim, Y.G.; Lee, J.H.; Raorane, C.J.; Oh, S.T.; Park, J.G.; Lee, J. Herring oil and omega fatty acids inhibit Staphylococcus aureus biofilm formation and virulence. *Front. Microbiol* **2018**, 9, 1241. DOI:10.3389/fmicb.2018.01241

41. Alonso, L.; Fraga, M.J.; Juarez, M. Determination of trans fatty acids and fatty acid profiles in margarines marketed in Spain. *J. Am. Oil Chem. Soc.* **2000**, 77(2), 131-136. DOI: 10.1007/s11746-000-0022-8

42. Silva, T.J.; Barrera-Arellano, D.; Ribeiro, A.P.B. Margarines: Historical approach, technological aspects, nutritional profile, and global trends. *Food Res. Int.* **2011**, 147, 110486. DOI: 10.1016/j.foodres.2021.110486

43. Garsetti, M.; Balentine, D.A., Zock, P.L.; Blom, W.A.; Wanders, A.J. Fat composition of vegetable oil spreads and margarines in the USA in 2013: a national marketplace analysis. *Int. J. Food Sci. Nutr.* **2016**, 67(4), 372-382. DOI: :10.3109/09637486.2016.1161012

44. Abramovič, H.; Vidrih, R.; Zlatić, E.; Kokalj, D.; Schreiner, M.; Žmitek, K.; Pravst, I.; et al. Trans fatty acids in margarines and shortenings in the food supply in Slovenia. *J. Food Compos. Anal.* **2018**, 74, 53-61. DOI: 10.1016/j.jfca.2018.08.007

45. CODEX, S. 33. Norma para los aceites de oliva y aceites de Orujo de oliva. Lima, Perú, 1981.

46. Paucar-Menacho, L.M.; Salvador-Reyes, R.; Guillén-Sánchez, J.; Capa-Robles, J.; Moreno-Rojo, C. Estudio comparativo de las características físico-químicas del aceite de sacha inchi (Plukenetia volubilis l.), aceite de oliva (Olea europaea) y aceite crudo de pescado. *Sci. Agropecu.* **2015**, 6(4), 279-290.

47. Škevin, D.; Kraljić, K.; Miletić, L., Obranović, M.; Neđeral, S.; Petričević, S. Adulteration of Oblica virgin olive oil with edible sunflower and refined olive pomace oil. *Croatian J. Food Technol. Biotechnol. Nutr.* **2011**, 6(3-4), 117-122.

48. Mateos, R.; Sarria, B.; Bravo, L. Nutritional and other health properties of olive pomace oil. *Crit. Rev. Food Sci. Nutr.* **2020**, 60(20), 3506-3521. DOI: 10.1080/10408398.2019.1698005

49. Allalout, A.; Krichène, D.; Methenni, K.; Taamalli, A.; Oueslati, I.; Daoud, D., & Zarrouk, M. Characterization of virgin olive oil from super intensive Spanish and Greek varieties grown in northern Tunisia. *Sci. Hortic.* **2009**, 120(1), 77-83. DOI: 10.1016/j.scienta.2008.10.006

50. Carrín, M.E.; Carelli, A. A. Peanut oil: Compositional data. *Eur. J. Lipid. Sci. Technol.* **2010**, 112(7), 697-707. DOI: 10.1002/ejlt.200900176

51. Yang, R.; Xue, L.; Zhang, L.; Wang, X.; Qi, X.; Jiang, J.; Li, P. et al. Phytosterol contents of edible oils and their contributions to estimated phytosterol intake in the Chinese diet. Foods **2019**, 8(8), 334. DOI: 10.3390/foods8080334

52. Ramos-Escudero, F.; Muñoz, A.M.; Ramos Escudero, M.; Viñas-Ospino, A.; Morales, M.T.; Asuero, A.G. Characterization of commercial Sacha inchi oil according to its composition: Tocopherols, fatty acids, sterols, triterpene and aliphatic alcohols. *J. Food Sci. Technol.* **2019**, 56(10), 4503-4515. DOI: 10.1007/s13197-019-03938-9

53. Matthaus, B.; Özcan, M.M.; Al Juhaimi, F.Y. Fatty acid composition and tocopherol profiles of safflower (Carthamus tinctorius L.) seed oils. *Nat. Prod. Res.* **2015**, 29(2), 193-196. DOI: 10.1080/14786419.2014.971316

54. CODEX, S. 329. Standard for Fish Oils. 2017.

55. Belhaj, N.; Arab-Tehrany, E.; Linder, M. Oxidative kinetics of salmon oil in bulk and in nanoemulsion stabilized by marine lecithin. *Process Biochem.* **2010**, 45(2), 187-195. DOI: 10.1016/j.procbio.2009.09.005

56. Suzuki, H.; Park, S.J.; Tamura, M.; Ando, S. Effect of the long-term feeding of dietary lipids on the learning ability, fatty acid composition of brain stem phospholipids and synaptic membrane fluidity in adult mice: a comparison of sardine oil diet with palm oil diet. *Mech. Ageing Dev.* **1998**, 101(1-2), 119-128. DOI: 10.1016/s0047-6374(97)00169-3

57. Gamez-Meza, N.; Higuera-Ciapara, I.; Calderon De La Barca, A.M.; Vázquez-Moreno, L.; Noriega-Rodriguez, J.; Angulo-Guerrero, O. Seasonal variation in the fatty acid composition and quality of sardine oil from Sardinops sagax caeruleus of the Gulf of California. *Lipids* **1999**, 34(6), 639-642.

58. Kheirati Rounizi, S.; Akrami Mohajeri, F.; Moshtaghi Broujeni, H.; Pourramezani, F.; Jambarsang, S.; Kiani, H.; Khalili Sadrabad, E. The chemical composition and heavy metal content of sesame oil produced by different methods: A risk assessment study. *Food Sci. Nutr.* **2021**, 9(6), 2886-2893. DOI: 10.1002/fsn3.2245

59. Ivanov, D.S.; Lević, J.D.; Sredanović, S.A. Fatty acid composition of various soybean products. *Food Feed Res.* **2010**, 37(2), 65-70.

60. Ruiz-Gutiérrez, V.; Pérez-Espinosa, A.; Vázquez, C.M.; Santa-Maria, C. (Effects of dietary fats (fish, olive and high-oleic-acid sunflower oils) on lipid composition and antioxidant enzymes in rat liver. *Br. J. Nutr.* **1999**, 82(3), 233-241. DOI: 10.1017/S0007114599001415

61. Xu, Z.; Ye, Z.; Li, Y.; Li, J.; Liu, Y. Comparative study of the oxidation stability of high oleic oils and palm oil during thermal treatment. *J. Oleo Sci.* **2020**, 69(6), 573-584. DOI: 10.5650/jos.ess19307

62. Romano, R.; Filosa, G.; Pizzolongo, F.; Durazzo, A.; Lucarini, M.; Severino, P.; Santini, A. et al. Oxidative stability of high oleic sunflower oil during deep-frying process of purple potato Purple Majesty. *Heliyon* **2021**, 7(3), e06294. DOI: 10.1016/j.heliyon.2021.e06294

63. Martínez, M.L.; Labuckas, D.O.; Lamarque, A.L.; Maestri, D.M. Walnut (Juglans regia L.): genetic resources, chemistry, by-products. *J. Sci. Food Agric.* **2010**, 90(12), 1959-1967. DOI: 10.1002/jsfa.4059

64. Copolovici, D.; Bungau, S.; Boscencu, R.; Tit, D.M.; Copolovici, L.J.R.C. The fatty acids composition and antioxidant activity of walnut cold press oil. *Rev. Chim.* **2017**, 68(3), 507-509.

65. Miraliakbari, H.; Shahidi, F. Antioxidant activity of minor components of tree nut oils. *Food Chem.* **2008**, 111(2), 421-427. DOI: 10.1016/j.foodchem.2008.04.008

66. Kamal-Eldin, A. Effect of fatty acids and tocopherols on the oxidative stability of vegetable oils. *Eur. J. Lipid Sci. Technol.* **2006**, 108(12), 1051-1061. DOI: 10.1002/ejlt.200600090

67. Hou, J.C.; Wang, F.; Wang, Y.T.; Xu, J.; Zhang, C.W. Assessment of trans fatty acids in edible oils in China. *Food Control* **2012**, 25(1), 211-215. DOI: 10.1016/j.foodcont.2011.10.044

68. Deen, A.; Visvanathan, R.; Wickramarachchi, D.; Marikkar, N.; Nammi, S.; Jayawardana, B.C.; Liyanage, R. Chemical composition and health benefits of coconut oil: an overview. *J. Sci. Food Agric.* **2021**, 101(6), 2182-2193. DOI: 10.1002/jsfa.10870

69. Hassanien, M.F.R. Tocol and phytosterol composition of edible oils in the Egyptian market. *J. Food Process. Preserv.* **2012**, 36(6), 531-538. DOI: 10.1111/j.1745-4549.2011.00621.x

70. El-Mallah, M.H.; El-Shami, S.M.; Hassanien, M.M.M.; Abdel-Razek, A.G. Effect of chemical refining steps on the minor and major components of cottonseed oil. *Agric. Biol. J. N. Am***.** **2011**, 2, 341-349.

71. Montserrat-de la Paz, S.; Fernández-Arche, Á.; Ángel-Martín, M.; García-Giménez, M.D. The sterols isolated from Evening Primrose oil modulate the release of proinflammatory mediators. *Int. J. Phytomedicine* **2012**, 19(12), 1072-1076. DOI: 10.1016/j.phymed.2012.06.008

72. Timoszuk, M.; Bielawska, K.; Skrzydlewska, E. Evening primrose (Oenothera biennis) biological activity dependent on chemical composition. *Antioxidants* **2018**, 7(8), 108. DOI: 10.3390/antiox7080108

73. Beveridge, T.H.; Girard, B.; Kopp, T.; Drover, J.C. Yield and composition of grape seed oils extracted by supercritical carbon dioxide and petroleum ether: varietal effects. *J. Agric. Food Chem.* **2005**, 53(5), 1799-1804. DOI: 10.1021/jf040295q

74. Azadmard-Damirchi, S. Review of the use of phytosterols as a detection tool for adulteration of olive oil with hazelnut oil. *Food Addit. Contam.* **2010**, 27(1), 1-10. DOI: 10.1080/02652030903225773

75. Szydłowska-Czerniak, A.; Trokowski, K.; Karlovits, G.; Szłyk, E. Effect of refining processes on antioxidant capacity, total contents of phenolics and carotenoids in palm oils. *Food Chem.* **2011**, 129(3), 1187-1192. DOI: 10.1016/j.foodchem.2011.05.101

76. Shad, M.A.; Pervez, H.; Zafar, Z.I.; Nawaz, H.; Khan, H. Physicochemical properties, fatty acid profile and antioxidant activity of peanut oil. *Pak. J. Bot.* **2012**, 44(1), 435-440.

77. Fanali, C.; Dugo, L.; Cacciola, F.; Beccaria, M.; Grasso, S.; Dacha, M.; Mondello, L.; et al. Chemical characterization of Sacha inchi (Plukenetia volubilis L.) oil. *J. Agric. Food Chem.* **2011**, 59(24), 13043-13049. DOI: 10.1021/jf203184y

78. Mohamed, H.M.A.; Awatif, I.I. The use of sesame oil unsaponifiable matter as a natural antioxidant. F*ood Chem.* **1998**, 62(3), 269-276. DOI: 10.1016/S0308-8146(97)00193-3

79. Aued-Pimentel, S.; Takemoto, E.; Antoniassi, R.; Badolato, E.S.G. Composition of tocopherols in sesame seed oil: an indicative of adulteration. *Grasas y aceites* **2006**, 57(2), 205-210. DOI: 10.3989/gya

80. Zeb, A.; Muhammad, B.; Ullah, F. Characterization of sesame (Sesamum indicum L.) seed oil from Pakistan for phenolic composition, quality characteristics and potential beneficial properties. *J. Food Meas. Charact.* **2017**, 11(3), 1362-1369. DOI:10.1007/s11694-017-9514-5

81. Dixit, S.; Das, M. Fatty Acid Composition Including Trans-Fatty Acids in Edible Oils and Fats: Probable Intake in Indian Population. *J. Food Sci.* **2012**, 77(10), T188-T199. DOI: 10.1111/j.1750-3841.2012.02875.x

82. De Greyt, W.; Radanyi, O.; Huyghebaert, A.; Kellens, M. Contribution of trans-Fatty Acids from Vegetable Oils and Margarines to the Belgian Diet. *Lipid/Fett* **1996**, 98(1), 30-33. DOI: 10.1002/lipi.19960980109
