# Supplementary material for: Virgin Olive Oil Ranks First in a New Nutritional Quality Score Due to Its Compositional Profile
Source: Nutrients. 2023 Apr 28;15(9):2127. doi: 10.3390/nu15092127 (PMC10180740; doi:10.3390/nu15092127)
Supplement: Supplementary file 1 [file nutrients-15-02127-s001.zip › Table S1.pdf]

Supplementary Table S1. Lowest, highest and mean content of the fatty acids included as criteria for the developing of the nutritional quality score of dietary oils (g/100g).

Oil	SFA			18:1			18:2			18:3			EPA+DHA		
	Lowest	Highest	Mean	Lowest	Highest	Mean	Lowest	Highest	Mean	Lowest	Highest	Mean	Lowest	Highest	Mean
**Almond** [1-4]	5.8	9.3	7.6	67.6	67.9	67.8	6.2	37.1	21.7	0.0	0.3	0.2	0.0	0.0	0.0
**Argan** [5-6]	15.8	23.1	19.5	43.0	49.1	46.1	27.2	39.3	33.3	0.0	0.5	0.3	0.0	0.0	0.0
**Avocado** [3,10-12]	11.6	31.7	21.7	58.4	76.5	67.5	9.0	17.5	13.3	0.3	3.2	1.8	0.0	0.0	0.0
**Beef tallow** [10,13, 14]	47.5	49.8	48.7	40.5	41.8	41.2	1.0	6.0	3.5	0.0	1.5	0.8	0.0	0.0	0.0
**Butter** [10,15]	50.5	67.5	59.0	23.4	29.4	26.4	1.8	2.3	2.1	0.4	1.2	0.8	0.0	0.0	0.0
**Camellia** [2,16,17]	17.2	17.3	17.3	72.5	79.8	76.2	7.5	9.5	8.5	0.3	0.6	0.5	0.0	0.0	0.0
**Canola** [1,2,10,18-21]	4.3	17.4	10.9	58.6	72.8	65.7	15.2	30.0	22.6	1.2	44.0	22.6	0.0	0.0	0.0
**Chia** [3,22-24]	9.3	14.0	11.7	6.0	11.0	8.5	11.7	20.4	16.1	60.2	65.2	62.7	0.0	0.0	0.0
**Coconut** [1,10,18-20, 25]	82.5	92.9	87.7	5.8	8.8	7.3	0.5	2.5	1.5	0.0	0.2	0.1	0.0	0.0	0.0
**Corn** [2,3,10,18-20]	13.4	25.1	19.3	24.8	38.7	31.8	34.0	65.6	49.8	0.0	2.0	1.0	0.0	0.0	0.0
**Cotton** [18-20,26,27]	10.5	28.2	19.4	17.5	35.4	26.5	42.0	64.3	53.2	0.0	0.4	0.2	0.0	0.0	0.0
**Evening primrose** [28-31]	8.7	13.9	11.3	7.2	11.5	9.4	61.0	73.1	67.1	8.7	12.9	10.8	0.0	0.0	0.0
**Flaxseed** [2,10,20, 32-33]	8.9	9.7	9.3	18.3	23.5	20.9	12.9	20.5	16.7	47.5	57.1	52.3	0.0	0.0	0.0
**Grapeseed** [10,12, 34-36]	9.7	12.6	11.2	14.5	22.2	18.4	58.0	78.0	68.0	0.0	1.1	0.6	0.0	0.0	0.0
**Hazelnut** [2,37,38]	7.2	16.7	12.0	74.5	83.2	78.9	7.6	16.6	12.1	0.0	0.2	0.1	0.0	0.0	0.0
**Herring** [14,39-40]	20.0	24.1	22.1	45.6	56.4	51.0	1.1	1.3	1.2	0.2	1.5	0.9	10.7	19.7	15.2

* EPD+DHA, ecosapentaenoic plus docosahexaenoic acid content; SFA, saturated fatty acids. For references, see References for Supplementary Tables S1 and S2.

Supplementary Table S1. (cont.). Lowest, highest and mean content of the fatty acids included as criteria for the developing of the nutritional quality score of dietary oils. (g/100g).

Oil	SFA			18:1			18:2			18:3			EPA+DHA (mg)		
	Lowest	Highest	Mean	Lowest	Highest	Mean	Lowest	Highest	Mean	Lowest	Highest	Mean	Lowest	Highest	Mean
**Lard** [10,13,14]	38.6	39.0	38.8	44.0	44.9	44.5	4.0	12.0	8.0	0.0	1.5	0.8	0.0	0.0	0.0
**Margarine** [41-44]	10.1	56.2	33.1	20.8	58.9	39.9	12.8	59.3	36.1	0.0	0.0	0.0	0.0	0.0	0.0
**Olive, common** [1,3,10,19,20,45,46]	15.3	20.7	18.0	66.6	80.9	73.8	3.5	21.0	12.3	0.0	1.6	0.8	0.0	0.0	0.0
**Olive, pomace** [19,45,47,48]	13.5	19.1	16.3	65.0	75.4	70.2	3.5	21.0	12.3	0.6	0.8	0.7	0.0	0.0	0.0
**Olive, Virgin** [10,16,45,49]	13.0	20.2	16.6	61.2	79.4	70.3	3.5	21.0	12.3	0.3	0.8	0.6	0.0	0.0	0.0
**Palm** [1,10,18,25]	46.3	49.5	47.9	37.0	41.5	39.3	9.0	12.0	10.5	0.0	0.5	0.3	0.0	0.0	0.0
**Peanut** [1,3,10,19,20,50,51]	12.6	19.2	15.9	38.6	74.2	56.4	18.2	43.2	30.7	0.0	0.3	0.2	0.0	0.0	0.0
**Sacha-inchi** [46,52]	6.8	15.9	11.4	9.7	24.7	17.2	21.3	53.6	37.5	10.1	49.0	29.6	0.0	0.0	0.0
**Safflower** [2,10,18,20,53]	7.2	9.8	8.5	11.6	16.6	14.1	54.3	83.2	68.8	0.0	0.2	0.1	0.0	0.0	0.0
**Salmon** [54,55]	23.8	23.8	23.8	35.5	35.5	35.5	1.5	15.0	8.3	0.0	6.0	3.0	5.0	25.5	15.3
**Sardine** [56,57]	31.9	31.9	31.9	24.4	24.4	24.4	1.6	1.8	1.7	0.4	0.8	0.6	23.1	32.7	27.9
**Sesame** [1,18,33,58]	16.9	18.6	17.8	40.6	42.0	41.3	40.1	47.9	44.0	0.2	0.8	0.5	0.0	0.0	0.0
**Soybean** [3,10,18-21,25]	12.2	23.9	18.1	16.4	38.1	27.3	47.6	59.0	53.3	4.5	12.1	8.3	0.0	0.0	0.0
**Sunflower** [1,3,10,18-21,25]	8.3	12.4	10.4	15.9	45.5	30.7	46.0	76.0	61.0	0.0	0.5	0.3	0.0	0.0	0.0
**Sunflower, high-oleic** [10,18,60-62]	7.8	9.9	8.9	83.7	85.2	84.4	2.1	17.0	9.6	0.0	0.3	0.2	0.0	0.0	0.0
**Walnut** [2,3,63,64]	8.0	12.3	10.2	14.0	26.3	20.2	53.7	63.2	58.4	0.1	14.4	7.2	0.0	0.0	0.0

* EPD+DHA, ecosapentaenoic plus docosahexaenoic acid content; SFA, saturated fatty acids. For references, see References for Supplementary Tables S1 and S2.
