# Supplementary material for: Virgin Olive Oil Ranks First in a New Nutritional Quality Score Due to Its Compositional Profile
Source: Nutrients. 2023 Apr 28;15(9):2127. doi: 10.3390/nu15092127 (PMC10180740; doi:10.3390/nu15092127)
Supplement: Supplementary file 1 [file nutrients-15-02127-s001.zip › Table S2.pdf]

Supplementary Table S2. Lowest, highest and mean content of trans fatty acids, tocopherols and sterols as criteria for the developing of the nutritional quality score of dietary oils.

Oil	TFA			Tocopherols			Phytosterols		
	Lowest	Highest	Mean	Lowest	Highest	Mean	Lowest	Highest	Mean
**Almond** [2-4,65]	0.0	0.0	0.0	0.0	119.8	59.9	47	455	251
**Argan** [5-6]	0.0	0.1	0.1	60.0	90.0	75.0	180	295	238
**Avocado** [3,12]	0.0	0.0	0.0	0.0	14.9	7.5	76	434	255
**Beef tallow** [10]	0.0	0.0	0.0	2.7	2.7	2.7	0	0	0
**Butter** [10]	3.0	3.0	3.0	2.3	2.3	2.3	0	4	2
**Camelia** [2,17,51]	0.0	0.0	0.0	52.2	52.2	52.2	35	143	89
**Canola** [2,10,14,21,37,51, 66,67]	0.1	3.8	2.0	43.0	268.0	155.5	327	1130	729
**Chia** [3,22-24]	0.0	0.0	0.0	23.8	47.6	35.7	455	455	455
**Coconut** [2,10,18,37,68]	0.0	0.0	0.0	0.0	0.5	0.3	40	120	80
**Corn** [2,3,10,18,37,51,66-69]	0.5	4.6	2.6	33.0	88.7	60.9	480	2300	1390
**Cotton** [18,27,66,70]	0.0	0.0	0.0	38.0	120.0	79.0	270	799	535
**Evening primrose** [28-31, 71,72]	0.0	0.0	0.0	26.3	34.1	30.2	971	1302	1137
**Flaxseed** [2,3,10,22,32,51, 66]	0.0	0.1	0.1	34.7	59.4	47.1	267	403	335
**Grapeseed** [10,34-36,51, 73]	0.0	0.0	0.0	24.0	42.4	33.2	200	1107	654
**Hazelnut** [3,37,38,65,74]	0.0	0.0	0.0	20.0	60.0	40.0	59	263	161
**Herring** [14,39]	0.0	0.0	0.0	0.4	8.1	4.3	0	0	0

* TFA, trans fatty acids. Values expressed as g/100g for TFA and mg/100g for tocopherols and sterols. For references, see References for Supplementary Tables S1 and S2.

Supplementary Table S2 (cont.). Lowest, highest and mean content of the fatty acids included as criteria for the developing of the nutritional quality score of dietary oils.

Oil	TFA			Tocopherols			Phytosterols		
	Lowest	Highest	Mean	Lowest	Highest	Mean	Lowest	Highest	Mean
**Lard** [10]	0.0	0.0	0.0	0.6	0.6	0.6	0	0	0
**Margarine** [41-44]	0.1	21.7	10.9	0.0	0.0	0.0	0	0	0
**Olive, common** [2,3,10, 48,51,66,68]	0.0	0.5	0.3	5.5	30.0	17.8	100	288	194
**Olive, pomace** [45,47,48]	0.0	0.8	0.4	18.5	30.0	24.3	180	300	240
**Olive, Virgin** [10,45,48, 49,68]	0.0	0.1	0.1	11.0	30.0	20.5	100	256	178
**Palm** [3,14,37,51,66,69, 75]	0.0	0.0	0.0	15.0	150.0	82.5	36	395	216
**Peanut** [3,22,37,50,51,76]	0.1	1.2	0.7	39.9	39.9	39.9	90	434	262
**Sacha-inchi** [52,77]	0.0	0.0	0.0	71.1	323.3	197.2	113	364	239
**Safflower** [2,10,18,37,53]	0.0	0.1	0.1	24.0	73.1	48.6	162	460	311
**Salmon** [54,55]	0.0	0.0	0.0	2.0	2.0	2.0	0	0	0
**Sardine** [56,57]	0.0	0.0	0.0	1.6	1.6	1.6	0	0	0
**Sesame** [3,18,37,51,78-80]	0.0	0.0	0.0	33.0	101.0	67.0	450	1900	1175
**Soybean** [14,22,50,51,67, 68,81,82]	0.2	3.6	1.9	60.0	337.0	198.5	180	900	540
**Sunflower** [1,3,10,18,21, 22,37,51,67-69,81,82]	0.1	1.8	1.0	44.0	152.0	98.0	205	500	353
**Sunflower, high-oleic** [10, 18,60,61]	0.0	0.0	0.0	52.4	52.4	52.4	170	520	345
**Walnut** [2,3,51,65]	0.0	0.0	0.0	14.9	34.7	24.8	90	283	187

* TFA, trans fatty acids. Values expressed as g/100g for TFA and mg/100g for tocopherols and sterols. For references, see References for Supplementary Tables S1 and S2.
